# Renal Primordia Activate Kidney Regenerative Events in a Rat Model of Progressive Renal Disease

**DOI:** 10.1371/journal.pone.0120235

**Published:** 2015-03-26

**Authors:** Barbara Imberti, Daniela Corna, Paola Rizzo, Christodoulos Xinaris, Mauro Abbate, Lorena Longaretti, Paola Cassis, Valentina Benedetti, Ariela Benigni, Carlamaria Zoja, Giuseppe Remuzzi, Marina Morigi

**Affiliations:** 1 IRCCS—Istituto di Ricerche Farmacologiche Mario Negri, Centro Anna Maria Astori, Science and Technology Park Kilometro Rosso, Bergamo, Italy; 2 Fondazione IRCCS—Policlinico San Matteo, Pavia, Italy; 3 IRCCS—Istituto di Ricerche Farmacologiche Mario Negri, Centro Ricerche Trapianti “Chiara Cucchi de Alessandri e Gilberto Crespi”, Ranica, Bergamo, Italy; 4 Unit of Nephrology and Dialysis, A.O. Papa Giovanni XXIII, Bergamo, Italy; University Medical Center Utrecht, NETHERLANDS

## Abstract

New intervention tools for severely damaged kidneys are in great demand to provide patients with a valid alternative to whole organ replacement. For repairing or replacing injured tissues, emerging approaches focus on using stem and progenitor cells. Embryonic kidneys represent an interesting option because, when transplanted to sites such as the renal capsule of healthy animals, they originate new renal structures. Here, we studied whether metanephroi possess developmental capacity when transplanted under the kidney capsule of MWF male rats, a model of spontaneous nephropathy. We found that six weeks post-transplantation, renal primordia developed glomeruli and tubuli able to filter blood and to produce urine in cyst-like structures. Newly developed metanephroi were able to initiate a regenerative-like process in host renal tissues adjacent to the graft in MWF male rats as indicated by an increase in cell proliferation and vascular density, accompanied by mRNA and protein upregulation of VEGF, FGF2, HGF, IGF-1 and Pax-2. The expression of SMP30 and NCAM was induced in tubular cells. Oxidative stress and apoptosis markedly decreased. Our study shows that embryonic kidneys generate functional nephrons when transplanted into animals with severe renal disease and at the same time activate events at least partly mimicking those observed in kidney tissues during renal regeneration.

## Introduction

Severely damaged kidneys possess limited regenerative potential. Therapeutic interventions are not sufficient to restore renal function in patients with end-stage renal disease. New alternative methodologies employing cellular therapies such as stem, renal precursor or adult cells, to rebuild or repair damaged renal structures are gaining increasing interest [1, 2]. A broad range of studies have shown that it is possible to use embryonic metanephroi to grow new kidneys in living hosts and integrate new filtering nephrons [3–8]. Kidney primordia implanted beneath the renal capsule or into tunnels in the cortices of host kidneys have integrated into living animals [5, 6]. Cultured fetal kidneys, upon *in vivo* transplantation, have also become vascularized and formed mature glomeruli [3]. The transplantation of xenogeneic renal primordium or metanephros, composed of embryonic kidney precursors programmed to develop into a kidney, is under investigation as a potential tool for supplying new functional renal cells [9].

Transplanting primordial tissues with an inherent ability to generate kidneys holds significant advantages over cell-based engineering approaches. Firstly, primordial tissues are already committed to generating a complete organ, alleviating the need to *de novo* assemble an intricate organ such as the kidney which is composed of structures of diverse embryological origin. Moreover, the side effects related to the use of stem cells, mainly maldifferentiation and tumorigenesis, are overcome. Grafting embryonic kidney precursors from xenogeneic sources offers immunological advantages compared with adult kidney transplants [10, 11].

Although a number of reports have demonstrated the survival and development of kidney anlagen in healthy hosts, the capacity of these primordia to grow in proximity of tissue injury remains largely unknown. Particularly, the success of transplanting the embryonic kidney beneath the kidney capsule may be affected by the hypoxic and proinflammatory microenvironment of the chronically injured kidney. These studies are highly relevant in view of the potential clinical application of this methodology in the treatment of chronic renal failure and they represent the fundamental prerequisite to its broad applicability. The aims of this study were twofold. Firstly we investigated whether embryonic kidneys, upon transplantation in syngeneic conditions under the kidney capsule in a rat model of progressive chronic nephropathy, exhibiting proteinuria, glomerulosclerosis, tubular damage and cast deposition [12–14], were able to survive, grow and develop into functional tissue. Moreover, we explored the possibility that metanephros implanted into a recipient with disease could activate in the host tissue the expression of genes and related proteins which are relevant to kidney regeneration. The latter hypothesis was also supported by evidence that some of the factors secreted during metanephros development [15, 16] may play a major part during kidney regeneration [17–19]. With this study we set a basis for the future applicability of the methodology of metanephros transplantation in kidneys affected by chronic disease providing new functional tissue as a local inducer of pro-regenerative processes.

## Materials and Methods

### Animals

Munich Wistar Frömter (MWF) inbred rats from our colony [13] (Charles River S.pA., Calco, Italy) were studied conforming with institutional guidelines in compliance with national and international laws and policies (EEC Council Directive 86/609, OJL 358, 1987; DL n116, G.U., suppl. 40, 18/2/1992, Circolare No.8, G.U., 14/7/1994; Guide for the Care and Use of Laboratory Animals, National Research Council, 1996). All animal studies were approved by the Institutional Animal Care and Use Committees of the Mario Negri Institute, Milan, Italy.

### Isolation and grafting of metanephroi

Time-mated MWF rats (gestational age E15) were sacrificed and embryos were surgically isolated. Metanephroi were dissected and placed in ice-cold Dulbecco’s modified medium (DMEM, Gibco, Life Technologies Italia, Monza MB, Italy) and immediately whole transplanted under the capsule of the left kidneys of 25 week-old male MWF rats. The sex of the embryos was not identified due to immediate transplantation. No immunosuppressive treatment was applied as we have transplanted metanephroi in syngeneic conditions.

### In vivo experimental design

We studied 2 groups of 25 week-old male MWF rats. Group MET: rats (n = 6) were transplanted with metanephroi (5 metanephroi per rat); Group Saline (n = 6). On the left kidney, a small incision was performed on the capsule and metanephroi or saline (200 μl) were administered underneath. Rats were sacrificed 6 weeks after transplantation (31 week-old) to determine renal function and histology. Semi-quantitative analyses of apoptosis, oxidative stress, proliferation, SMP30 expression and morphometric analysis of RECA-1 were performed in areas adjacent to the graft (adjacent: within about 500 μm from the periphery inside the cortex, along the extension of the graft) and in distant areas (distant: about 500 μm far from the graft). VEGF, FGF2, HGF, IGF-1 and Pax-2 expression was quantified in areas adjacent to the graft and representative images for each marker were acquired in adjacent fields. The observed effects of grafts on adjacent host renal tissue were found and measured within two high power fields (HPF) from the kidney capsule.

In additional experiments, female MWF rats (n = 3) were transplanted under the kidney capsule with E15 syngeneic metanephroi (n = 5) following the same experimental procedure of male rats. Other MWF female rats (n = 3) received saline. Animals were sacrificed 6 weeks after grafting.

As controls, to test the effect of other cell types, 25 week-old male MWF rats were grafted with skin fibroblasts and sacrificed 6 weeks later (n = 3/each group). Fibroblasts were isolated from skin biopsies of MWF syngeneic rats and used at first passage after *in vitro* expansion. After trypsin detachment, 1x10^6^ fibroblasts were transplanted under the kidney capsule.

### Renal function and serum creatinine measurement

Urinary concentration of total proteins (n = 4/group for male and n = 3/group for female rats) was measured using the Coomassie method with a Cobas Mira autoanalyzer (Roche Diagnostic Systems, Basel, Switzerland). Serum creatinine (n = 3/group) was measured with Cobas Mira autoanalyzer. Creatinine in urine and in cysts (n = 3/group) was measured using the alkaline picrate method (Creatinine companion, Exocell, Philadelphia, USA).

### FITC-labeled albumin perfusion

FITC-labeled bovine serum albumin (FITC-BSA, Sigma-Aldrich, Milan, Italy), 5 mg in 0.5 ml saline, was intravenously injected 30 min before sacrificing the animals (n = 3/group). Frozen sections of Periodate-Lysine-Paraformadehyde (PLP)-fixed kidneys were prepared and further stained with Rhodamine-labeled Lens Culinaris Agglutinin (LCA, Vector laboratories, Burlingame, CA, USA) and 4’,6’-diamidino-2-phenylindole dihydrochloride hydrate (DAPI, Sigma-Aldrich) to improve the identification of structures for analysis.

### Renal histology

Duboscq-Brazil fixed kidney samples were stained with hematoxylin and eosin, or periodic acid-Schiff’s reagent (PAS). Glomerulosclerosis was assessed in non-overlapping fields (up to 20 for each section, n = 3/group). Each glomerulus was scored according to the extension of sclerotic changes as follows: 0 = absence of sclerosis; 1 = sclerotic changes affecting less than 25% of glomerular tuft area; 2 and 3 = lesions affecting 25 to 50% and more than 50 to 75% of the tuft, and 4 = lesions exceeding 75% of the tuft. The average glomerulosclerosis index was calculated using the weight-average of each class. Luminal hyaline casts and tubular necrosis (denudation of the tubular basement membrane) were assessed in up to 10 non-overlapping fields (40x, HPF) for each section, both in areas adjacent to and distant from the graft.

### Volume density (Vv) of endothelium and length density (Jv) of peritubular capillaries

PLP-fixed kidneys were stained with anti-rat endothelial cell antigen 1 (MCA970R, RECA-1, AbD Serotec, Kidlington, Oxford, UK) followed by goat anti-mouse Cy5 (Jackson Immunoresearch, West Grove, PA, USA) and counterstained with FITC-WGA (Vector Laboratories) and DAPI (Sigma-Aldrich). To estimate the area density occupied by RECA-1 staining, 3 renal sections/rat were digitized. For each section, 20 fields were acquired in areas adjacent to and distant from the graft. Each image (512x512 pixels) was digitally overlapped with an orthogonal grid composed of 600 points. Vv and Jv were calculated as previously reported [20].

### Oxidative stress and apoptosis

Oxidative damage was evaluated through protein nitration of tyrosine residues by using rabbit polyclonal anti-nitrotyrosine (Upstate Biotechnology, Billerica, MA, USA). At least 20 non-overlapping sequential fields (n = 3 rats/group) were analyzed in areas adjacent to and distant from the grafts. Positive tubuli/HPF were counted.

Apoptosis was measured by TUNEL assay (Roche Mannheim, Germany). Apoptotic nuclei and DAPI positive cells per field were counted. For each section (n = 3 sections/rat, n = 3 rats/group), 15 fields were acquired in areas adjacent to the graft and in non-adjacent areas.

### Proliferation

Proliferating tubular cells were identified by monoclonal antibody against the proliferating cell nuclear antigen Ki-67 (MCL-Ki67-MM1, Novocastra Laboratories, Newcastle, UK). Slides were counterstained with Rhodamine-LCA (Vector laboratories) and DAPI (Sigma-Aldrich). Proliferating cells were counted in up to 20 fields per animal in areas adjacent to and distant from the graft.

### Immunostaining studies

PLP-fixed kidney tissues were employed for immunofluorescence studies. The following primary antibodies were used: rabbit polyclonal anti WT1 antibody (sc-192, Santa Cruz Biotechnology Inc., Santa Cruz, CA), mouse monoclonal anti RECA-1 antibody (MCA970R, AbD Serotec), goat polyclonal anti megalin antibody (sc-16478, Santa Cruz Biotechnology), mouse monoclonal anti FGF2 antibody (610073, BD BioScience, San Diego, CA), rabbit polyclonal anti HGF antibody (sc-7949, Santa Cruz Biotechnology), goat polyclonal anti IGF-1 antibody (sc-1422, Santa Cruz Biotechnology), rabbit polyclonal anti Pax-2 antibody (Life Technologies Italia) and monoclonal antibody anti NCAM (Developmental Studies Hybridoma Bank, University of Iowa), polyclonal rabbit antibody anti CD31 (Abcam, Cambridge, UK), monoclonal rabbit antibody anti Ki-67 (Abcam). To quantify SMP30 positive tubuli, sections were stained with goat anti rat SMP30 (sc-25951, Santa Cruz Biotechnology) and rhodamine-labeled LCA (Vector laboratories) and DAPI (Sigma-Aldrich). Positive tubuli were counted in up to 20 fields in 3 sections for each animal (n = 4 rats/group). For VEGF staining, Duboscq-Brazil fixed sections were incubated with goat anti-rat VEGF antibody (AF564, R&D Systems, Minneapolis, MN, USA). Infiltrating cells were quantified by immunostaining of ED-1 positive cells (MAB1435, AbDSerotec, Kidlington, UK) and the number of ED-1 positive cells were counted per field (n = 15 fields/animal) in n = 3 animals per group.

VEGF, FGF2, HGF, IGF-1 and Pax-2 signals were graded on a scale from 0 to 3 related to the expression of the markers in each HPF (0 = no signal, 1 = weak, 2 = mild, 3 = diffuse; n = 3 rats/group). To evaluate tubulogenesis and glomerulogenesis in metanephroi transplated in male and female rats, the number of tubuli and synaptopodin-positive glomeruli was quantified for each HPF (n = 3 rats/group). Vasculogenesis in the graft was assessed by giving a semiquantitative score of RECA-1 expression to each HPF (0 = no signal, 1 = weak, 2 = mild, 3 = diffuse; n = 3 rats/group).

### Quantitative Real Time-PCR (qRT-PCR)

Total RNA was extracted from kidneys using TRIzol reagent (Invitrogen, Life Technologies Italia) according to the manufacturer’s instructions. Contaminating genomic DNA was removed by RNase-free DNase (Promega, Ingelheim, Germany) for 1h at 37°C. Two μg of purified RNA were reverse transcribed using random hexamers oligonucleotides and 50U of SuperScript II RT (Invitrogen, Life Technologies Italia) for 1h at 42°C. No enzyme was added for reverse transcriptase-negative controls. The PCR was performed on the 7300 Real Time PCR System (Applied Biosystems, Life Technologies Italia). The amplification profile consisted of 2 min at 50°C and 10 min at 95°C, the samples were cycled 40 times at 95°C for 15 s and 60°C for 60 s. We used the 2-ΔΔ Ct technique. Fold change in mRNA expression was calculated based on cycle threshold (Ct) differences between treated MWF rats and MWF rats given only saline (calibrator). The sequence of primers used for qRT-PCR is shown in Table 1.

**Table 1:** Primers used for Real Time RT-PCR of rat HGF, IGF-1, VEGF, FGF2, Pax-2 and 18S.

Target	Sequence
HGF	for 5’—GAATGCATGACCTGCAACGG -3’
	rev 5’—TGTCGGGATATCTTTCCGGC -3’
IGF-1	for 5’—GCTTTTACTTCAACAAGCCCACA -3’
	rev 5’—TCAGCGGAGCACAGTACATC -3’
VEGF	for 5’—AACGAAAGCGCAAGAAATCC -3’
	rev 5’—GCTCACAGTGAACGCTCCAG -3’
FGF2	for 5’—GAACCGGTACCTGGCTATGA -3’
	rev 5’—CCGTTTTGGATCCGAGTTTA -3’
Pax-2	for 5’—CAAAGTTCAGCAGCCTTTCC -3’
	rev 5’—GTTAGAGGCGCTGGAAACAG -3’
18S	for 5’—ACGGCTACCACATCCAAGGA -3’
	rev 5’—CGGGAGTGGGGTAATTTGCG-3’

### Statistical analysis

The results are expressed as mean ± SE. Data were analyzed using ANOVA followed by Bonferroni, or T-Test, as appropriate. Non-parametric data were analyzed using the Kruskal-Wallis test. Statistical significance level was defined as *P* < 0.05.

## Results

### Metanephroi develop and connect to the host when transplanted under the kidney capsule of rats with progressive nephropathy

**Fig 1:**
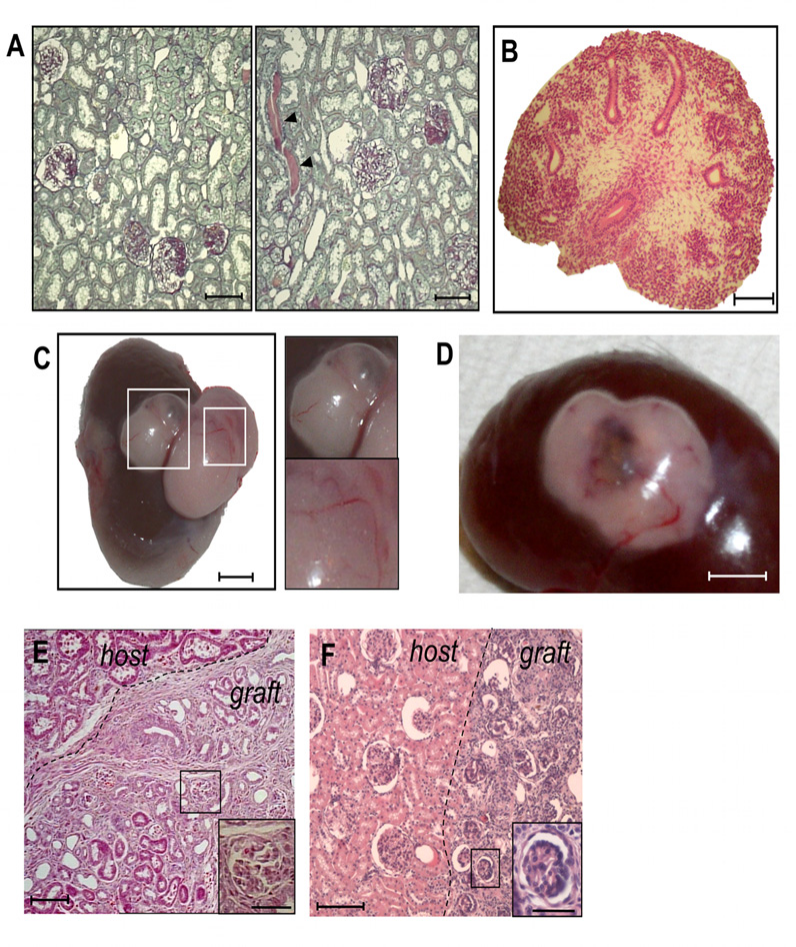
Histological appearance of adult male and female MWF rat kidneys and E15 MWF rat metanephros. **(A)** Representative images of kidney tissues in untreated male MWF rats at 25 (left) and 31 (right) weeks of age. Arrowheads indicate tubular casts. Sections were stained with PAS. Scale bars = 100 μm. **(B)** Overall appearance of E15 rat metanephros (MET) stained with haematoxylin and eosin. Scale bar = 100 μm. **(C)** Kidney explanted from male MWF rat, six weeks after MET transplantation, showing metanephroi grown into large structures with fluid-containing cysts. Scale bar = 2.5 mm. Enlargements highlight cyst and vascularization. **(D)** Macroscopic view of kidney from female MWF rat grafted with MET, six weeks after transplantation. Scale bar = 2.5 mm. **(E)** Renal histology (haematoxylin and eosin staining) of male MWF rat grafted with MET and sacrificed after 6 weeks from transplant. Scale bars = 100 μm. High magnification of a representative glomerulus developed in the graft is shown in the inset. Scale bar = 50 μm. **(F)** Histology of renal tissue from female MWF rat transplanted with MET and sacrificed after 6 weeks from transplant. Scale bar = 100 μm. The inset reports a representative glomerulus developed in the graft. Scale bar = 50 μm.

**Fig 2:**
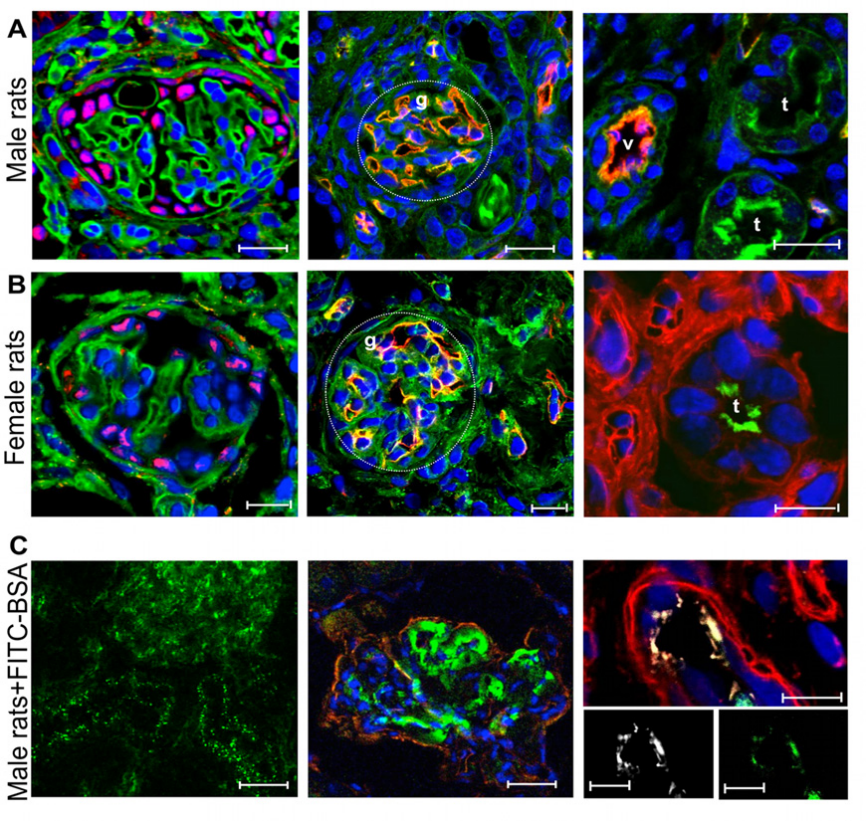
Vascularization and growth of renal structures in the transplanted metanephroi and intragraft albumin uptake in male MWF rat transplanted with metanephroi. **(A)** Immunofluorescence detection of WT-1 and rat endothelial cell marker (RECA-1) in metanephroi (MET) developed in male MWF rats. On the left: glomerulus stained for WT1 (red). Scale bar = 10 μm. Center: capillaries of a glomerulus (g) labeled for RECA-1 (red). Scale bar = 20 μm. Right: RECA-1 positive vessels (v, red) and tubuli (t). Scale bar = 20 μm. WGA lectin (green) was used to display renal structures and DAPI (blue) to visualize nuclei. **(B)** Metanephroi developed in female MWF rats. On the left: glomerulus stained for WT1 (red), FITC-labeled WGA lectin (green) and DAPI (blue). Center: section labeled for rat endothelial cell marker RECA-1 (red), FITC-labeled WGA lectin (green) and DAPI (blue). Right: proximal tubule stained for megalin (green), rhodamine-labeled LCA lectin (red) and DAPI (blue). Scale bars = 10 μm. **(C)** Localization of FITC-BSA (green) intravenously injected in MET-transplanted male recipient and graft. Host tissues (left), glomerulus (center) and megalin-positive tubule (white, right) of the graft. The tissue was also visualized by labeling with rhodamine-labeled LCA lectin (red) and DAPI (blue). Scale bars = 20 μm (left and center) and 10 μm (right).

The possibility of employing the transplantation of the embryonic kidney—the metanephros—as a therapeutic tool for severely damaged kidneys depends on the capacity of the kidney anlagen to develop in a chronic renal failure setting. To test whether a pathological environment would allow metanephros development, we transplanted embryonic kidneys under the kidney capsule of syngeneic male MWF rats, a model of progressive nephropathy and, as a control, in their healthy counterparts, syngeneic female MWF rats [14]. We studied the effect of grafted metanephroi in 25 week-old MWF male rats that exhibited established proteinuria, glomerulosclerosis (Table 2) and sparse protein casts and tubular enlargement (Fig. 1A), as previously reported [12]. Urinary protein excretion progressively increased with age associated with worsening of glomerulosclerosis (Table 2) and increase of cast frequency and tubular damage (Fig. 1A). Serum creatinine did not increase between 25 and 31 weeks (Table 2). At the time of transplantation under the kidney capsule of syngeneic male MWF rats, E15 metanephroi obtained from MWF embryos contained mesenchyme and rudimentary epithelial structures but no glomeruli, as expected (Fig. 1B). After 6 weeks, metanephroi underwent remarkable growth and developed in macroscopically visible structures, with vascularization and cyst-like structures containing fluid (Fig. 1C). When metanephroi were transplanted in healthy female MWF rats, the outgrowth of macroscopically visible vascularized tissue containing cyst-like structures was also supported (Fig. 1D). As shown in Fig. 1E and 1F, grafts were easily localized by position and morphology compared to the host tissues. Histological analysis revealed that metanephroi had undergone nephrogenesis, as evidenced by the presence of glomeruli and tubuli (Fig. 1E and 1F). No significant differences were noted between primordia developed in male or female animals in terms of number of glomeruli (numbers/40x microscopic field, male vs female host: 3.72 ± 0.14 vs 4.92 ± 0.90), tubuli (numbers/40x microscopic field, male vs female host: 18.98 ± 1.51 vs 18.09 ± 4.57) or blood vessels (score 0–3, male vs female host: 2.08 ± 0.12 vs 2.12 ± 0.12). Developing glomeruli contained cells positive for the podocyte marker WT1 (Fig. 2A and 2B, left) and a well-organized capillary tuft highlighted by RECA-1 staining (Fig. 2A and 2B, center).

**Table 2:** Glomerulosclerosis, proteinuria and creatinine in male MWF rats pre- and post-metanephros transplantation or saline treatment.

	MWF	MWF+SALINE	MWF+MET
	*(25 wks)*	*(31 wks)*	*(31 wks)*
**Glomerulosclerosis**	0.42 ± 0.04	1.86 ± 0.3*	1.8 ± 0.4*
**Index**			
**Proteinuria**	276 ± 24	463 ± 53*	566 ± 27*
*(mg/24h)*			
**Serum creatinine**	0.5 ± 0	0.53 ± 0.02	0.51 ± 0.01
*(mg/dl)*			
**Urine creatinine**	111 ± 10	146.88 ± 6.1	93.22 ± 16.18
*(mg/dl)*			
**Cyst creatinine**	-	-	24.09 ± 4.9^#^
*(mg/dl)*			

Degree of glomerulosclerosis, proteinuria and creatinine in male MWF rats pre- (25 wks) and post- (31 wks) metanephros (MET) or saline treatment. Creatinine concentration in cysts of MWF animals receiving MET. Urine creatinine is also shown.

*P < 0.05 vs 25 wks,

^#^P < 0.05 vs urine creatinine of rats receiving MET at 31 wks.

Tubuli exhibited well-developed brush borders and apical expression of megalin (Fig. 2A, 2B and 2C, right). Small and medium-sized capillaries contained RECA-1 positive endothelium and supported the vascularization of the parenchyma (Fig. 2A, right).

In order to investigate whether developed metanephroi were functionally vascularized and connected to the host, we studied the distribution of intravenously-injected FITC-BSA in male MWF rats. FITC-BSA was localized in host tissues (Fig. 2C, left) but also reached the graft where it was found in vessels and in the capillary tuft of the glomeruli (Fig. 2C, center). Moreover, the fluorescent BSA signal was present in tubular structures of the graft, identified using megalin staining, suggesting tubuli were capable of protein uptake (Fig. 2C, right). No signal due to autofluorescence was observed in saline-injected animals not receiving FITC-BSA. Functional parameters related to cyst-like structures generated from the grafted metanephroi were then analyzed. Cysts (Fig. 1C and 1D) contained fluid in a volume range of 10–15 μl. Creatinine concentration in the cyst fluid was higher compared to serum (Table 2) while it was lower compared to the urine of animals that received metanephroi (Table 2).

To assess whether the presence of damage in the host affects the production of growth factors of the transplanted renal primordia, the expression of HGF, IGF-1 and FGF2 was compared using immunohistochemical analysis in grafted metanephroi after their transplantation in male and female recipients. Statistically significant increases were found for HGF and FGF2 in grafts given to male hosts, whereas a tendency to increase was observed for IGF-1 (score 0–3, male vs female, HGF: 1.56 ± 0.1 vs 1.04 ± 0.21, P < 0.05; FGF2: 1.81 ± 0.43 vs 0.7 ± 0.28, P < 0.01; IGF-1: 2.00 ± 0.08 vs 1.37 ± 0.55).

### Effect of transplanted metanephroi on renal functional parameters, glomerulosclerosis and fibrosis

Male MWF rats at 6 weeks after metanephros transplantation exhibited proteinuria, and serum and urine creatinine values similar to those observed in saline-treated rats (Table 2). Overall, the glomerulosclerosis index at 31 weeks was not reduced by metanephros transplantation. To assess the effect of grafted metanephroi on interstitial fibrosis, the expression of genes such as collagen 1 A2, collagen 3 and alpha-SMA was analysed. In animals that received metanephros transplants, mRNA expression of collagen 1 A2, (MET, 0.7 ± 0.05 vs Saline, 1), collagen 3 (MET, 1.08 ± 0.04 vs Saline, 1) and alpha SMA (MET, 1.1 ± 0.025 vs Saline, 1) was comparable to that observed in the saline group.

### Transplanted metanephroi influence and improve recipient kidney vascularization

**Fig 3:**
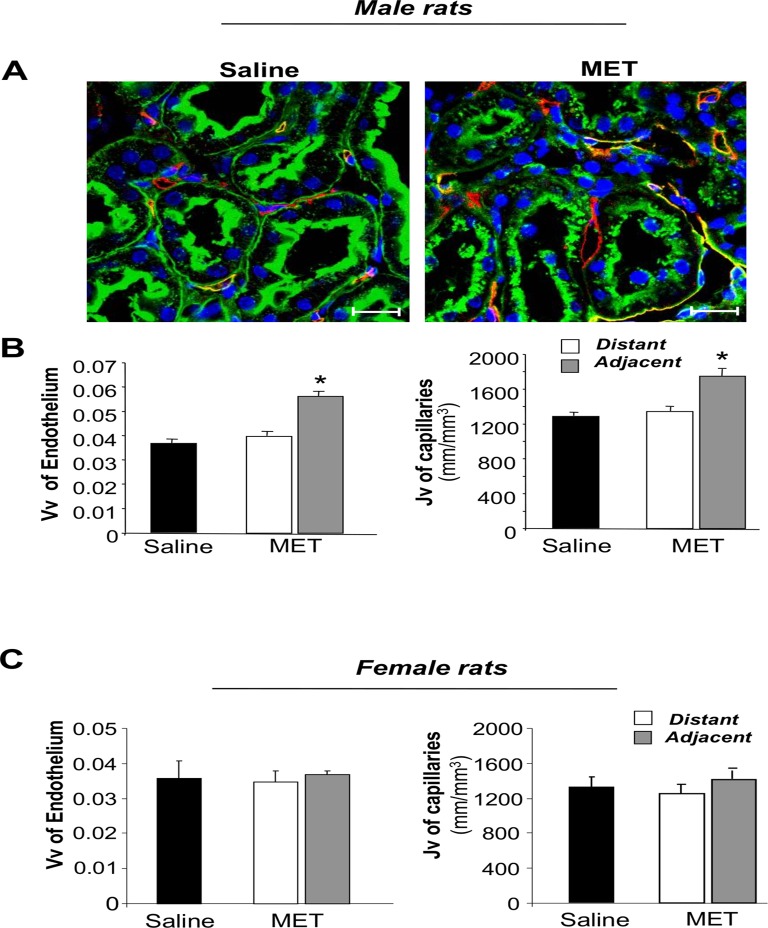
Metanephros effect on endothelium volume density and peritubular capillary length density. **(A)** Representative images of renal tissue of male MWF rats receiving saline or methanephroi (MET), 6 weeks after transplantation, stained for RECA-1 (red), FITC-labeled WGA lectin (green) and DAPI (blue); Scale bars = 20 μm. **(B-C)** Endothelial volume density (Vv, on the left), and length density of peritubular capillaries (Jv, on the right), evaluated in renal tissues of male **(B)** and female **(C)** animals receiving saline or MET distant from or adjacent to the graft. The results are expressed as mean ± SE. *P < 0.01 *vs* respective adjacent tissues and *vs* saline (n = 4 rats/group).

Capillary rarefaction and reduced blood supply are common features of chronic renal disease [21]. In order to study the impact of transplanted metanephroi on the regeneration of host renal tissues, the vascularization in areas adjacent to the graft was quantified. Kidney sections of male MWF rats receiving saline or metanephroi were labeled with an antibody specific to rat endothelium (RECA-1) (Fig. 3A). Using morphometric analysis, endothelium volume density (Vv) of peritubular capillaries of 31 week-old MWF rats significantly increased (41% increase) compared to areas distant from the metanephroi (Fig. 3B). The length density of peritubular capillaries (Jv) in areas adjacent to the graft significantly increased (28% increase) compared to distant ones in animals grafted with metanephroi (Fig. 3B). A representative image, at low magnification, showing the area affected by vascular changes in grafted male animals is supplied in S1 Fig. In female MWF, the development of metanephroi did not affect either peritubular capillary Vv or Jv of recipient kidneys in areas adjacent to or distant from the graft (Fig. 3C).

### Transplanted metanephroi reduce renal oxidative damage and apoptosis in host kidneys

**Fig 4:**
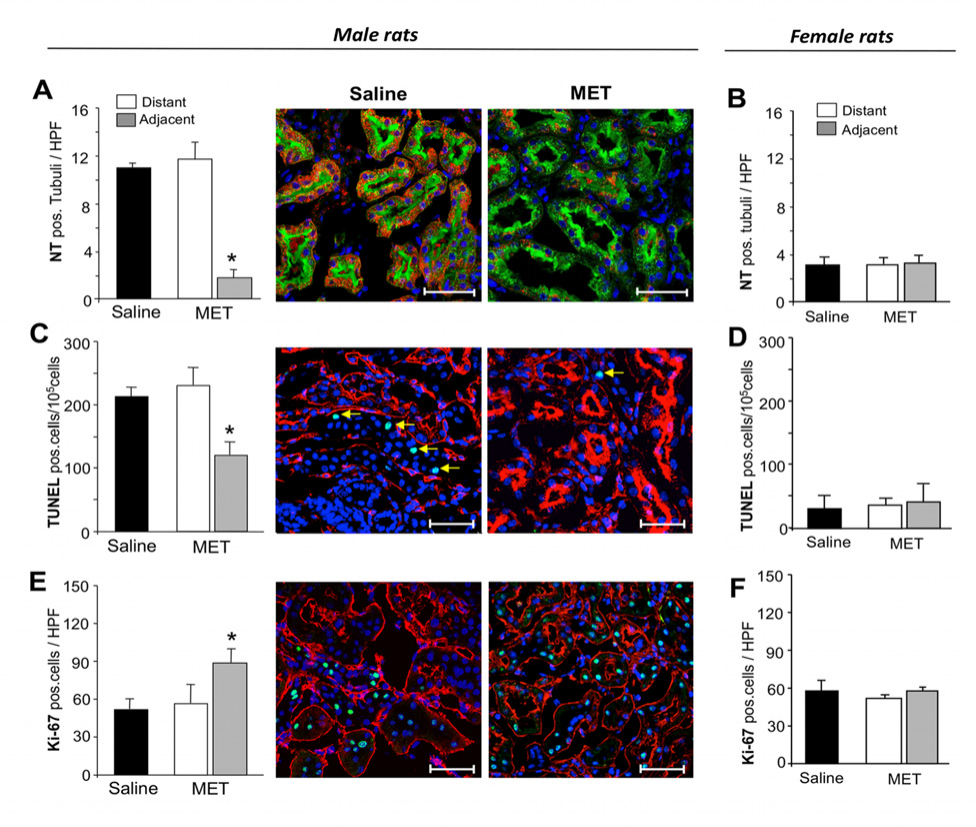
Metanephros effect on oxidative damage, apoptosis and proliferation of recipient renal tissues. (**A)** Oxidative damage was quantified in renal tissues of male animals receiving saline or methanephroi (MET), after 6 weeks, as percentage of tubuli positive for nitrotyrosine (NT), in fields distant from or adjacent to the grafts. Data are expressed as mean ± SE. *P < 0.05 *vs* areas distant from the graft and *vs* saline (n = 3 rats/group). Pictures show renal tissues of male MWF rats receiving saline or MET stained for nitrotyrosine (red), FITC-labeled lectin WGA (green) and DAPI (blue). Scale bars = 50 μm. (**B)** Quantification of oxidative damage in female animals receiving saline or MET, in fields distant from or adjacent to the grafts. Data are expressed as mean ± SE (n = 3 rats/group). (**C**) Saline or MET-treated male animals were evaluated for the presence of apoptotic cells by counting the number of cells positive for TUNEL in fields adjacent to or distant from the graft. Data are expressed as mean ± SE. *P < 0.05 *vs* areas distant from the graft and *vs* saline (n = 3 rats/group). Immunofluorescence images show host renal tissues stained with TUNEL (green), rodhamine-LCA lectin (red) and DAPI (blue), obtained from saline or MET-treated animals. Arrows indicate apoptotic nuclei. Scale bars = 50 μm. (**D)** Quantification of apoptotic cells in female animals receiving saline or MET, in fields distant from or adjacent to the grafts. Data are expressed as mean ± SE (n = 3 rats/group). (**E)** Quantification of Ki-67 positive cells in renal tissues of male animals treated with saline or MET after 6 weeks, in fields distant or adjacent to the grafts. Data are expressed as mean ± SE. *P < 0.05 *vs* areas distant from the graft and *vs* saline (n = 5 rats/group). Pictures show representative fields of host renal tissues of male MWF rats receiving saline or MET showing Ki-67 positive nuclei (green). Tissues are also stained with rodhamine-LCA lectin (red) and DAPI (blue). Scale bars = 50 μm. (**F)** Quantification of proliferating cells in female animals receiving saline or MET, in fields distant from or adjacent to the grafts. Data are expressed as mean ± SE (n = 3 rats/group).

Oxidative stress plays a crucial role in worsening chronic kidney injury. Peroxynitrite, the reaction product of nitric oxide with superoxide anion, has been identified as the key oxidant species involved in the direct nitration of tyrosine residues causing protein oxidation. In 31 week-old male MWF rats administered saline, we observed the presence of a high number of tubuli positive for nitrotyrosine staining (Fig. 4A). In areas adjacent to the grafted metanephroi a dramatic reduction in oxidative stress occurred compared to distant regions of the same kidney (Fig. 4A). The same area experienced improved vascularization (S2 Fig.).

In the context of chronic progressive disease, apoptosis contributes to renal damage leading to tubular atrophy. In areas adjacent to metanephroi, a reduction of the number of TUNEL positive cells was observed compared to distant fields (Fig. 4C). In healthy female MWF rats treated with saline, renal cell oxidative stress and apoptosis was very scarce and did not change upon MET transplantation (Fig. 4B and D).

### Metanephros transplantation induces cell proliferation in recipient renal tissues

Regeneration of host renal tissues was investigated by studying the Ki-67 nuclear marker of cell proliferation. An augmentation of tubular cell proliferation was found in areas adjacent to the grafts compared to comparable distant areas (Fig. 4E). Transplantation with metanephroi induced a significant increase in cell division in host tissue (Fig. 4E). A higher presence of proliferating cells was found in the areas with improved vascularization in grafted male animals (S2 Fig.). When we analyzed renal tissues of healthy female MWF rats with metanephros transplants, cell proliferation was found unchanged in areas adjacent to or distant from the graft, exhibiting similar values to those observed in female animals receiving saline (Fig. 4F).

### Metanephros transplantation induces expression of markers relevant to kidney regeneration

**Fig 5:**
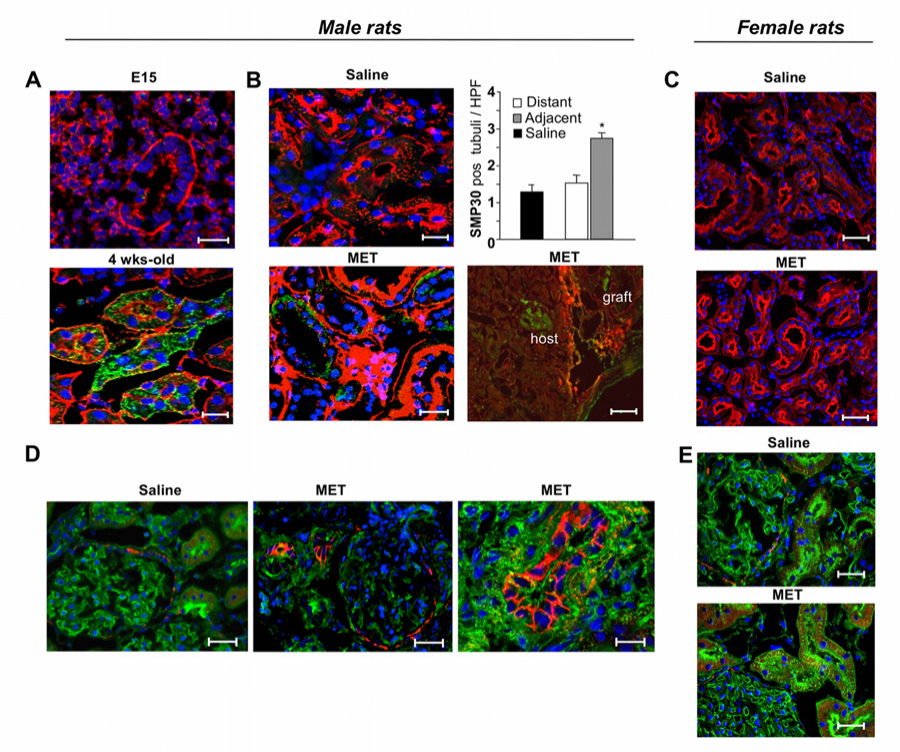
Effect of transplanted metanephroi on SMP30 and NCAM expression in recipient renal tissues. (**A)** Representative images of SMP30 expression (green) in renal male tissues stained with rhodamine-labeled LCA lectin (red) and DAPI (blue). In metanephros (MET), analyzed at the time of transplantation (E15), no SMP30 expression was present, whereas in 4 week-old rat kidney it was abundantly expressed. Scale bars = 25 μm. **(B)** At 31 weeks of age, no staining for SMP30 was observed in host tissue of MWF rats receiving saline. In contrast, SMP30 positive tubuli were present in host tissue of MWF rats that received a MET transplant. Lower magnification image is provided in right panel where increased expression of SMP30 in areas adjacent to MET is shown. Scale bars = 25 μm; = 100 μm in right panel. Histogram shows quantification of SMP30-positive tubuli in renal tissues of male animals that received a MET transplant or saline, after 6 weeks. Data are expressed as mean ± SE; *P < 0.05 *vs* areas distant from the graft and *vs* saline (n = 4 rats/group). **(C)** Representative pictures of SMP30 staining in recipient renal tissue of female animals receiving saline or MET transplant. Scale bars = 50 μm. **(D)** The expression of NCAM (red) in renal tissues of saline or MET-treated male MWF rats is shown on renal sections stained with FITC-labeled WGA-lectin (green) and DAPI staining (blue). Higher magnification image is provided in right panel. Scale bars = 20 μm; = 10 μm in right panel. **(E)** Representative pictures of NCAM staining in recipient renal tissue of female animals receiving saline or MET transplant. Scale bars = 20 μm.

In order to understand the mechanisms underlying metanephros-dependent induction of vasoprotective, angiogenic and mitogenic effects counterbalanced by a decrease in renal oxidative stress and apoptosis, we investigated factors recognized to be involved in early developmental/regenerative processes [22–25]. First we studied the expression of senescence marker protein 30 (SMP30), a protein normally expressed in maturing but not in adult tissues, described as possessing anti-apoptotic and anti-oxidative effects [26, 27]. SMP30 was not expressed in metanephroi at the time of transplantation, but it was abundantly present in tubular cells of 4 week-old kidneys (Fig. 5A), as previously reported [26]. In MWF male rats treated with saline at 31 weeks, SMP30 was not detected (Fig. 5B). In contrast, metanephroi transplantation induced a significant increase in SMP30 expression in tubular host cells in focal areas adjacent to the grafts compared to distant ones (positive tubuli/HPF, adjacent *vs* distant areas: 2.75 ± 0.15 *vs* 1.54 ± 0.21; P < 0.05) (Fig. 5B). SMP30 staining was completely absent in healthy female rats receiving saline or MET (Fig. 5C).

Moreover, we investigated the expression of NCAM, a molecule expressed both in the embryonic kidney and in tubuli of adult renal tissues during recovery from acute ischemia [28, 29]. NCAM is a potential renal progenitor cell marker in rats, where NCAM-positive glomerular cells co-express the progenitor marker CD24 [30] and also in human fetal kidneys, where nephron progenitors have been isolated using NCAM immunosorting [31]. In the renal tissues of male MWF rats receiving saline, NCAM was expressed by glomerular parietal cells (Fig. 5D), as previously reported [30], and by scattered cells in tubuli. Several NCAM-positive tubuli were present in focal areas of the cortex adjacent to the graft in the renal tissues of rats with metanephroi transplants (Fig. 5D). In healthy female rats receiving saline or metanephroi, NCAM-staining was observed in glomerular parietal cells (Fig. 5E) and in rare cells in tubuli.

**Fig 6:**
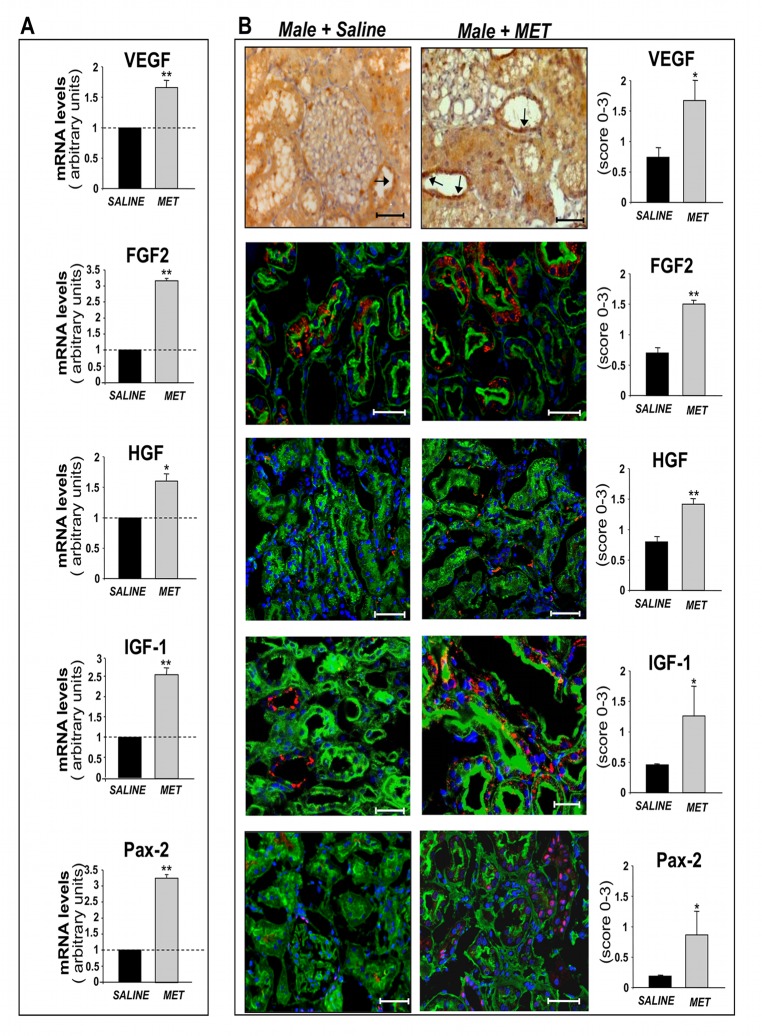
Expression of mRNAs and proteins relevant to kidney regeneration in male MWF rats. **(A)** Real Time RT-PCR analysis of VEGF, FGF2, HGF, IGF-1 and Pax-2 in whole host renal tissues of male MWF rats receiving saline or metanephroi (MET) (in adjacent area), 6 weeks after transplantation. Data are expressed as mean ± SE; *P < 0.05, **P < 0.01 *vs* saline (n = 4 rats/group, VEGF n = 6 rats/group). (**B)** Immunohistochemical staining of VEGF (arrows) in male animals receiving saline or MET transplant, after 6 weeks. Scale bars = 50 μm. Representative immunofluorescence stainings (red) of FGF2, HGF, IGF-1 and Pax-2 in rats receiving saline or MET, on renal tissues labeled with WGA-lectin (green) and DAPI (blue). Scale bars = 50 μm, = 25 μm (for FGF2 and IGF-1). Semiquantitative analysis of immunohistochemical staining of VEGF, FGF2, HGF, IGF-1 and Pax-2 in male MWF rats receiving saline or MET. Data are expressed as mean ± SE; *P < 0.05, **P < 0.01 *vs* saline (n = 3/group).

**Fig 7:**
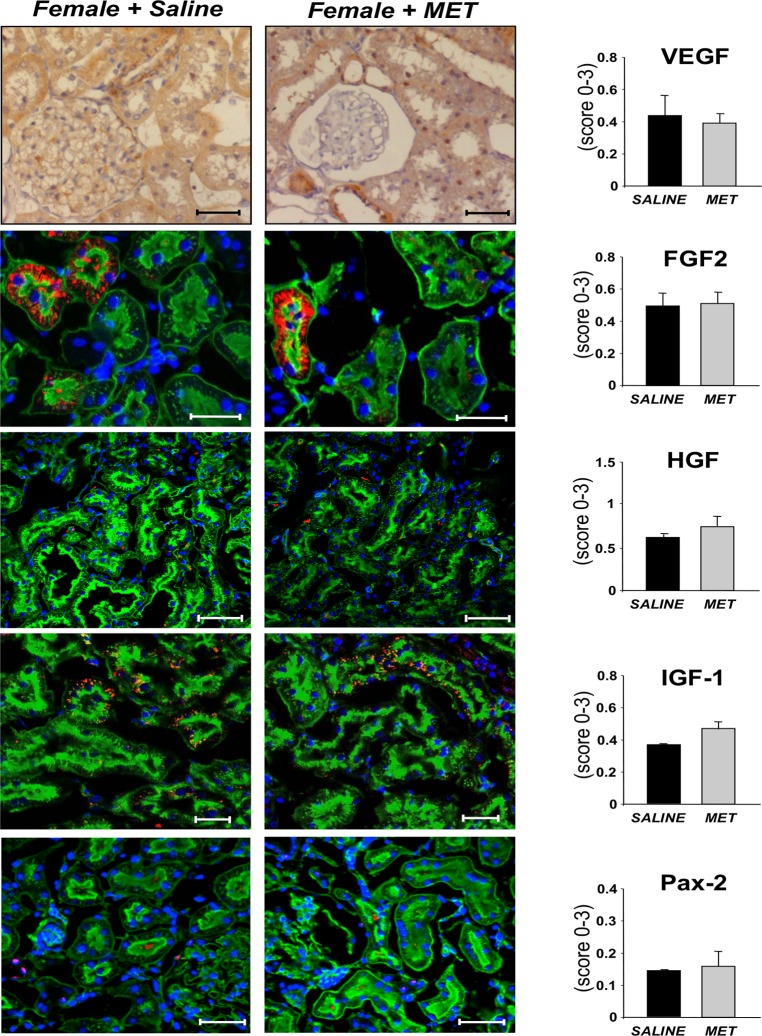
Expression of proteins relevant to kidney regeneration in female MWF rats. Immunohistochemical staining of VEGF in female animals receiving saline or MET transplant, after 6 weeks. Scale bars = 50 μm. Representative immunofluorescence staining (red) of FGF2, HGF, IGF-1 and Pax-2 in female rats receiving saline or MET, on renal tissues labeled with WGA-lectin (green) and DAPI (blue). Scale bars = 50 μm, = 25 μm (for FGF2 and IGF-1). Semiquantitative analysis of immunohistochemical stainings of VEGF, FGF2, HGF, IGF-1 and Pax-2 in female MWF rats receiving saline or MET. Data are expressed as mean ± SE (n = 3/group).

The expression of several genes and related proteins relevant to kidney regeneration [22–25, 32] was studied in host renal tissues of animals receiving metanephroi in the adjacent areas compared to saline. A remarkable increase in mRNA expression of growth factors including VEGF, FGF2, HGF, IGF-1 was observed in renal tissues rats with metanephros transplants compared to saline (Fig. 6A). Moreover, transcription factor Pax-2 was significantly upregulated (Fig. 6A). Consistent with mRNA expression data, immunohistochemical analysis confirmed that protein expression of the growth factors clearly increased in renal tissues adjacent to MET compared to saline (Fig. 6B and S1 Fig.), the same area that was associated with improved vascularization (S2 Fig.). Moreover, Pax-2 was expressed in several tubuli of metanephros-graft animals whereas they were rare or absent in saline-treated rats (Fig. 6B). In contrast, in female MWF rats we did not observe significant metanephros-induced changes in protein expression of any of the growth or transcription factors studied (Fig. 7). Both in male and female animals, we have not observed immunohistochemical differences in the expression of growth/transcription factors between renal tissues of animals receiving saline or MET in areas distant from the graft (not shown).

### ED-1 positive cells do not increase in the renal tissue of grafted animals

To exclude the possibility that infiltrating monocytes/macrophages in the host renal tissue might have increased as a consequence of the presence of the graft and might have influenced the host tissue’s response, we stained sections with ED-1 antibody. Animals receiving saline or metanephroi showed no difference in the number of ED-1-positive cells in all the samples analyzed (ED-1 positive cells/field, saline *vs* metanephroi, 80.6 ± 4.5 *vs* 79 ± 4.6).

### Effect of fibroblast transplantation on male MWF kidneys

**Fig 8:**
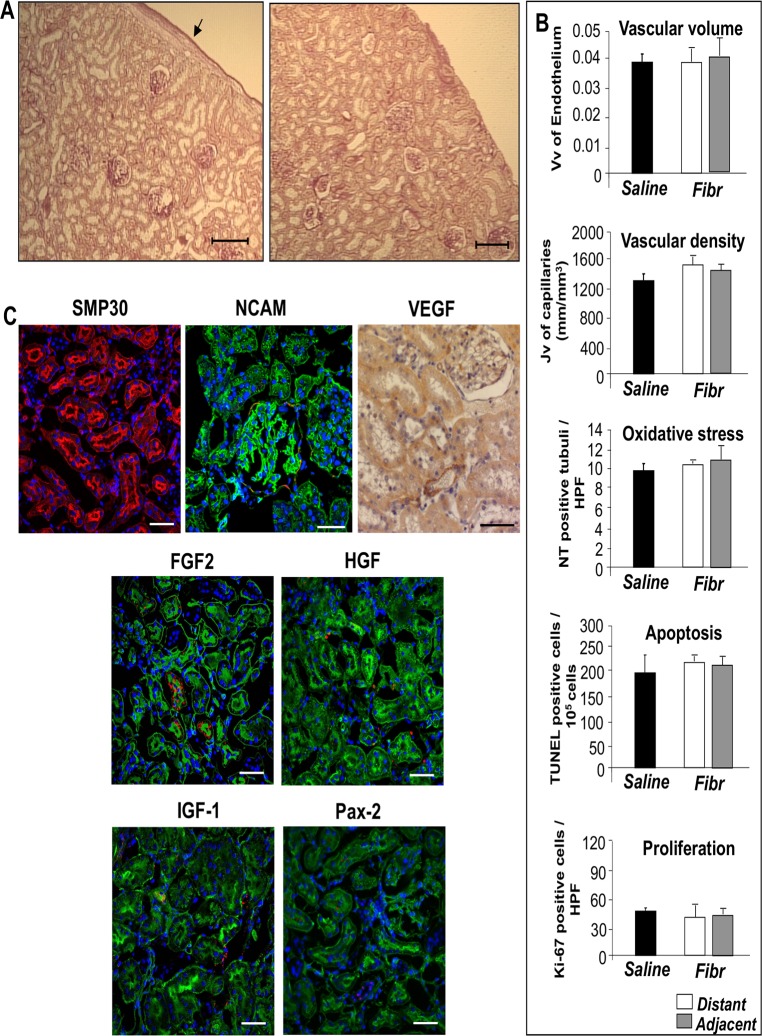
Effect of fibroblasts transplanted under the kidney capsule of male MWF rats. (**A**) Histological appearance of renal tissue from male MWF rat transplanted with fibroblasts in adjacent (left, arrow) or distant (right) area. Scale bars = 200 μm. (**B**) Quantification of endothelium volume density (Vv), peritubular capillary length density (Jv), nitrotyrosine (NT)-positive tubuli, TUNEL-positive cells and Ki-67-positive cells, in renal tissues of MWF rats receiving fibroblasts or saline. Data are expressed as mean ± SE (n = 3 rats/group). (**C**) Representative immunohistochemical images of SMP30 (green), NCAM (red), VEGF (brown signal), FGF2 (red), HGF (red), IGF-1 (red) and Pax-2 (red) in male rats with fibroblast transplant. Sections are co-stained with rhodamine-LCA (in SMP30 panel, red) or with FITC-WGA lectin (in panels showing NCAM, FGF2, HGF, IGF-1, Pax2, green) and DAPI (blue). Scale bars = 50 μm.

In order to document whether the effect of the grafts observed on host renal tissue is distinctive for metanephroi, we transplanted syngeneic skin fibroblasts under the kidney capsule of male MWF 25 week-old rats. Animals receiving fibroblasts did not show improvement of renal damage (Fig. 8A), oxidative stress or apoptosis (Fig. 8B). Similarly, vascularization and tubular cell proliferation were not affected by fibroblast transplantation either in the area adjacent to or distant from the graft, which were comparable to the saline group (Fig. 8B). In parallel, no change in protein expression of growth and transcription factors was observed (Fig. 8C).

## Discussion

New technologies based on tissue engineering approaches are enlivening the challenge to find a solution for the treatment of severely injured organs. The approaches of developing new kidneys starting with either renal primordia transplanted under the kidney capsule or organoids obtained from an embryonic renal cell suspension are promising methodologies [7, 8, 10, 33]. However, it was not clear to which extent the environment of chronically injured tissue of the host could influence graft development. In end-stage renal failure, reduced vascularization deriving from chronic hypoxia is associated with severe oxidative damage and fibrosis [34] which may hamper anlagen sprouting. Our present study goes a step further towards what has been reported in healthy animals [4, 5] and proves that metanephroi transplanted under the kidney capsule of rats with progressive kidney disease undergo successful organogenesis with development into functional nephrons. In MWF rats, a model of progressive nephropathy, developed grafts are functionally active and can primitively produce urine as suggested by the higher concentration of creatinine in the cyst-like structure formed in the graft compared to levels in the blood. Well-formed glomerular capillary tufts and small vessels of the graft are connected with host circulation as proved by the presence of systemically injected fluorescein-labeled BSA in vessels and capillary tufts of neo-kidneys. Moreover, fluorescent BSA is found in tubular structures of the graft, showing the capacity of tubuli to absorb labeled protein. Altogether these data indicate that chronically injured kidneys allow the development of grafted metanephroi and have the potential to establish functional interactions with the graft.

The second aim of the present study was to assess whether the anlagen could establish a beneficial interaction with the recipient renal tissue so as to activate promising processes for tissue regeneration. The fact that the implanted metanephroi did locally ameliorate the perfusion of host renal tissue was indicated by data showing increased length and volume density of peritubular capillaries in MWF male rat kidneys. Importantly, increased renal vascularization was accompanied by evidence of increased tubular cell proliferation in host tissue in the vicinity of metanephroi and by the reduction of oxidative stress and apoptosis suggesting that the renal tissue is proceeding toward repair. In the search for mediators capable of activating a local regenerative process within the host renal tissue, we focused on growth and transcription factors known to be relevant to kidney regeneration [22–25]. Studies documented that a wide range of differentially expressed nephrogenic proteins, including FGF2 and Pax-2, were induced during renal regeneration after hypoxia [25]. FGF2 treatment ameliorated renal functional and structural damage in experimental chronic kidney disease [19]. Moreover, growth factors like HGF or IGF-1 showed therapeutic potential in animal models of acute kidney injury through multiple actions that may concur to create a pro-regenerative environment [17, 24, 32, 35, 36]. HGF effectively enhanced regenerative repair by exerting mitogenic and anti-apoptotic actions on renal tubular and glomerular cells [24]. Accelerated recovery from acute kidney injury using IGF-1 treatment was mediated by both increases in glomerular blood flow and tubular cell proliferation, and inhibition of apoptosis [32, 35–37]. Among the major proangiogenic growth factors, VEGF is critically compromised in progressive renal disease [18, 38–40]. In the remnant kidney model, VEGF treatment reduced fibrosis and stabilized renal function [39], while in pigs undergoing renal artery stenosis, VEGF modulated glomerular and tubular cytokine expression, thereby improving microvascular density and renal function [41]. In the present study, the evidence from Real Time-PCR and immunohistochemical analysis that the recipient kidneys exhibited concomitant upregulation of Pax-2, FGF2, HGF, IGF-1 and VEGF in areas adjacent to metanephroi, may suggest that a pro-regenerative response in the damaged tissue is activated in the presence of grafted metanephros, mimicking some of the cellular events observed in kidney tissues during renal regeneration. It is likely that the upregulation of growth factors under this condition in the host contributed to the enhanced vascularization and tubular cell proliferation that we detected in the same areas adjacent to the graft.

The active role of the transplanted kidney anlagen in eliciting host responses is underlined by the finding of intragraft upregulation of molecules such as HGF and FGF2 upon anlagen transplantation within the diseased host. Lower expression levels of the same factors were found within the graft in control female hosts, thus suggesting that the renal primordia could sense damage in the host with disease, enhancing the intragraft synthesis of growth factors. The precise mechanisms and mediators underlying this host-graft cross-talk are elusive. Oxidative stress or hypoxia, as we have detected in the host, might be the triggering events. Finally, primordia can affect the recipient damaged renal tissue both through direct paracrine action and by enhancing the endogenous expression of growth factors, a possibility supported by recognized evidence of reciprocal induction between mediators including HGF, IGF-1 and VEGF [42, 43].

The pro-regenerative potential of the graft is additionally suggested by the induction of SMP30 in tubular cells of male MWF rats in the proximity of metanephroi. SMP30 is both expressed during tissue maturation, and decreased with aging [26] and it is involved in the protection of tubular cells against oxidative injury and apoptosis [27, 44]. Its induction in the tubular cells, together with the appearance of NCAM-positive cells, could suggest recapitulation of the tubular cell developmental program, as happens during the recovery phase from acute injury for NCAM and other relevant molecules [25, 28, 45].

The favorable interactions induced by anlagen transplantation and the formation of pre-urine in graft-developed cysts were not accompanied by significant improvement in renal functional parameters in terms of proteinuria and serum creatinine. The protocol we used here was not designed primarily to assess therapeutic effects on renal function, which indeed were not expected to become evident to a considerable extent. Possible limitations could be partly related to the fact that only one of the kidneys received the graft and that the area of implantation was confined to a restricted portion of the organ. Increasing the number of metanephroi and their transplantation sites might overcome both the lack of significant functional effects in the presence of regeneration, and the fact that no reductions in the degrees of glomerulosclerosis and interstitial fibrosis could be disclosed. One might also expect that a longer period of treatment could enhance the therapeutic benefits of such an approach, particularly in terms of chronic renal structural damage.

Altogether, our results suggest that the development of new renal structures in transplanted primordia, in the setting of chronic renal injury, is possible and also activates local responses which are reminiscent of tissue regeneration, by virtue of effective cross-talk between host and graft tissues. With the present study, a new point of view on the use of the embryonic kidney has been highlighted in the context of forefront treatments of renal disease. Traditionally, the transplantation of metanephroi has been investigated with the final aim of creating new functional renal units that might work in place of dysfunctional kidneys. Here, the emerging concept relies on the capacity of renal anlagen to reactivate, within the host kidney with established disease, pathways that locally promote its own regeneration. This concept holds promises for the development of regenerative strategies based on the characterization and provision of the appropriate milieu responsible for beneficial effects. Conceiving new devices to support *in vivo* development of renal xenogeneic tissues as a source of regenerative factors for the time period necessary for recovery may represent a novel intriguing challenge for regenerative medicine by means of tissue engineering approaches.

## Supporting Information

S1 Fig Low magnification immunofluorescence staining (red) for RECA-1, HGF, IGF-1 and Pax-2 on male MWF renal tissues. Sections are labeled with WGA-lectin (green) and DAPI (blue). Dashed lines divide the graft from the host, while dotted lines divide the area adjacent to and distant from the graft. Scale bars = 100 μm.
(TIF)

S2 Fig Co-staining of vascular endothelium with markers relevant to kidney regeneration in male MWF rats receiving metanephroi (MET). Double immunofluorescence staining of endothelial cell markers (RECA-1 or CD31) with oxidative damage marker nitrotyrosine (NT), proliferation marker Ki-67 and HGF, IGF-1 or Pax-2. Improved vascularization in areas adjacent to MET is associated with improvement of oxidative damage and increased expression of the markers associated with regeneration. Renal tissues are labeled with DAPI (blue) and where specified in the picture with WGA-lectin. Scale bars = 100 μm.
(TIF)
