# Supplementary figures and images for: Renal Primordia Activate Kidney Regenerative Events in a Rat Model of Progressive Renal Disease
Source: PLoS One. 2015 Mar 26;10(3):e0120235. doi: 10.1371/journal.pone.0120235 (PMC4374877; doi:10.1371/journal.pone.0120235)

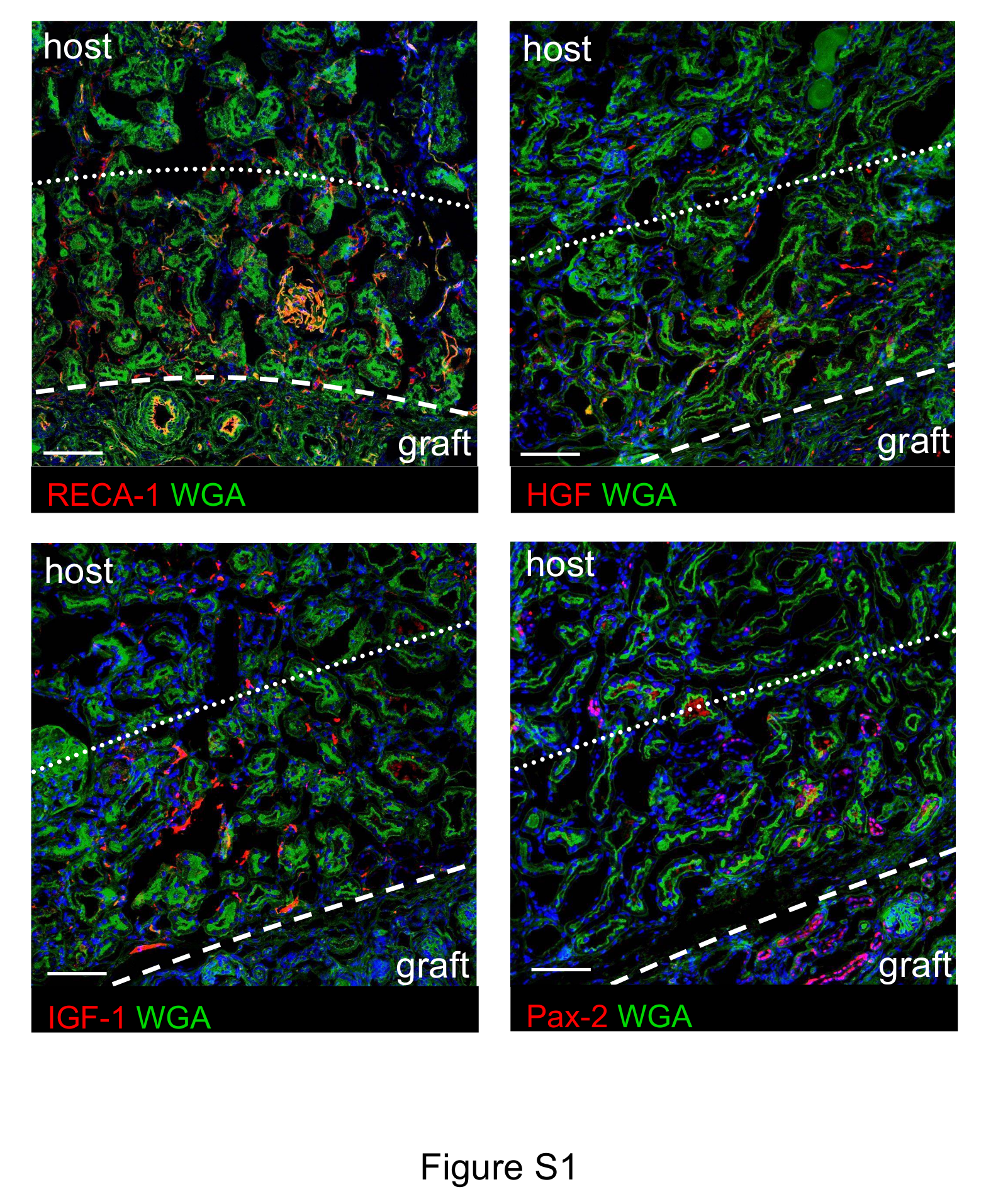

Supplement: S1 Fig — Sections are labeled with WGA-lectin (green) and DAPI (blue). Dashed lines divide the graft from the host, while dotted lines divide the area adjacent to and distant from the graft. Scale bars = 100 μm. (TIF) [file pone.0120235.s001.tif]

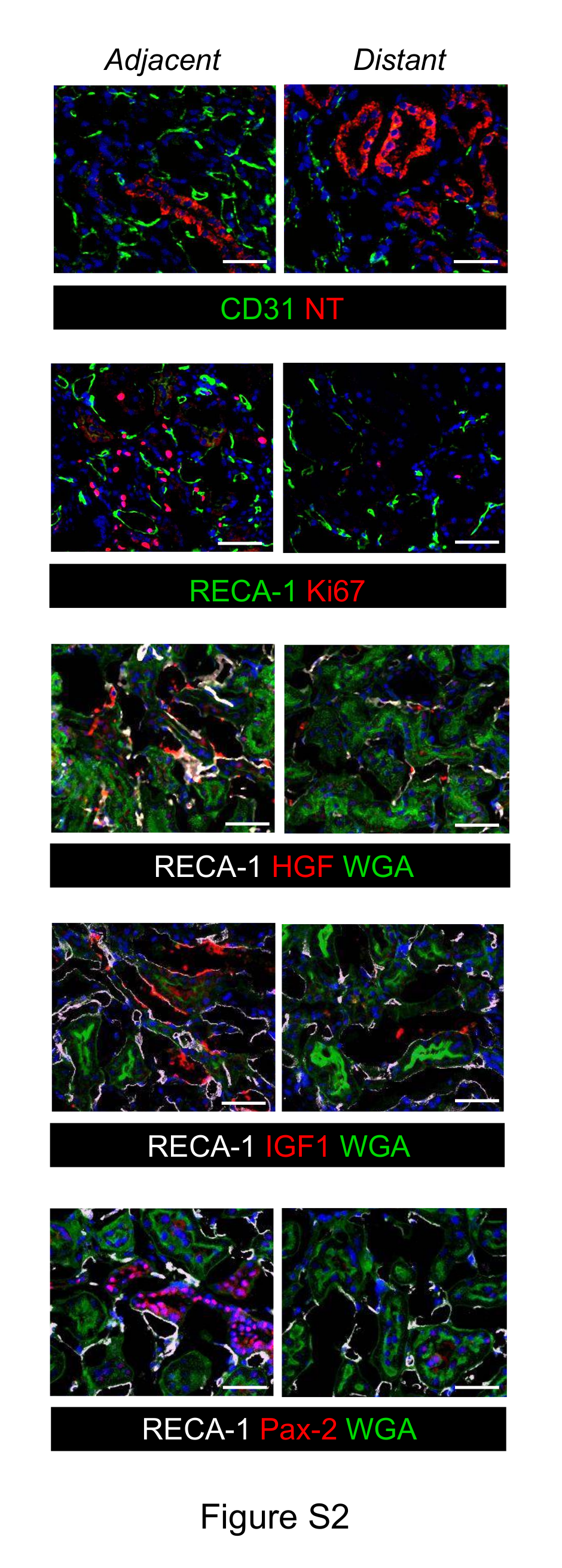

Supplement: S2 Fig — Double immunofluorescence staining of endothelial cell markers (RECA-1 or CD31) with oxidative damage marker nitrotyrosine (NT), proliferation marker Ki-67 and HGF, IGF-1 or Pax-2. Improved vascularization in areas adjacent to MET is associated with improvement of oxidative damage and increased expression of the markers associated with regeneration. Renal tissues are labeled with DAPI (blue) and where specified in the picture with WGA-lectin. Scale bars = 100 μm. (TIF) [file pone.0120235.s002.tif]
